# Adaptive Staircase Measurement of Hand Proprioception

**DOI:** 10.1371/journal.pone.0135757

**Published:** 2015-08-14

**Authors:** Najmeh Hoseini, Brandon M. Sexton, Karl Kurtz, Yang Liu, Hannah J. Block

**Affiliations:** 1 Physical Therapy Program, Midwestern University, Glendale, Arizona, United States of America; 2 Department of Kinesiology, Indiana University Bloomington, Bloomington, Indiana, United States of America; 3 Department of Occupational Therapy, Florida International University, Miami, Florida, United States of America; 4 Program in Neuroscience, Indiana University Bloomington, Bloomington, Indiana, United States of America; The University of Western Ontario, CANADA

## Abstract

Clinicians and researchers often need to measure proprioception (position sense), for example to monitor the progress of disease, to identify the cause of movement or balance problems, or to ascertain the effects of an intervention. While researchers can use sophisticated equipment to estimate proprioceptive acuity with good precision, clinicians lack this option and must rely on the subjective and imprecise methods currently available in the clinic. Here we describe a novel technique that applies psychometric adaptive staircase procedures to hand proprioception with a simple tablet-style apparatus that could easily be adapted for the clinic. We report test-retest reliability, inter-rater reliability, and construct validity of the adaptive staircase method vs. two other methods that are commonly used in clinical settings: passive motion direction discrimination (PMDD) and matching. As a first step, we focus on healthy adults. Subjects ages 18–82 had their proprioception measured with each of the three techniques, at the metacarpophalangeal joint in the second finger of the right hand. A subset completed a second session in which the measures were repeated, to assess test-retest reliability. Another subset had the measurements done by two different testers to assess inter-rater reliability. Construct validity was assessed using stepwise regression on age and activity level, and correlations calculated across the three methods. Results suggest that of the three methods, the adaptive staircase method yields the best test-retest reliability, inter-rater reliability, and construct validity. The adaptive staircase method may prove to be a valuable clinical tool where more accurate assessment of proprioception is needed.

## Introduction

To interact efficiently with our environment, for example reaching to pick up a mug, we need a sense of where our hand is and how it is moving. This arises from the proprioceptive senses, including static position sense, movement sense or kinesthesia, and sense of force or heaviness, among others [1]. Proprioception is critical for accurate movement, but is frequently impaired following stroke [2, 3]. This has important functional consequences [4], including poor recovery of mobility [5], ability to function in daily activities [6], and impaired motor learning [7]. Proprioceptive deficits are thought to play a role in the motor impairments associated with other conditions as well, including multiple sclerosis [8], Parkinson’s and Huntington’s diseases [9], fall risk in the elderly [10, 11], concussion [12], autism [13, 14], and chronic pain [15, 16]. Hand/finger proprioception is particularly important because of the role it plays in manual dexterity and associated tasks of daily living.

To manage therapy plans and reevaluate patients after interventions, clinicians often must assess proprioceptive acuity [17]. One common clinical test is passive movement direction discrimination (PMDD), which measures movement sense: the joint is passively extended or flexed and the subject must report the perceived direction [18–21]. In a matching test, the subject actively moves the testing joint to match the reference joint on the opposite side of the body; the closeness of the match is thought to reflect static position sense. However, these and other available clinical tests are subjective, poorly standardized, and too coarse to detect subtle changes [21, 22]. In addition, these tests do not control for the influence of muscle contraction history on proprioceptors (spindles) in the muscle, i.e. muscle thixotropy, which can bias proprioceptive measurements [1, 21]; and they require active or passive movement of the patient, creating a confound for patients with pain, spasticity, or motor deficits. For example, a patient with pain may tense up with movement, providing extra stimulation to spindles and leading to over-estimation of proprioceptive acuity. The patient may have less pain on a return visit, yielding a more accurate proprioceptive estimate but erroneously indicating a decrease in acuity since the first visit.

An additional limitation of current clinical tests is that proprioceptive sensitivity and bias are not distinguished. If a series of numerical measurements of proprioception is made, the spread of the subject’s errors reflects the inverse of his proprioceptive sensitivity. If the mean of the errors is offset from true position, this offset represents a bias in perception. Sensitivity and bias are independent; in other words, proprioception can be very sensitive but highly biased. Perceptual sensitivity is thought to be represented in the brain in a Bayesian fashion [23], but sensory bias is less understood. It is possible that some clinical populations have a deficit in proprioceptive sensitivity while others have difficulties because their proprioception is biased. It would thus be advantageous to develop a test that incorporates both sensitivity and bias, making it possible to tailor rehabilitation to the specific proprioceptive deficit.

Although clinical tests of static position sense, such as matching, involve a movement before each trial, this need not be the case. Activity in a population of muscle spindle afferents does not stop when we stop moving. Background spindle activity continues, which, along with joint receptors and skin stretch input, the brain integrates into a body map to create a perception of position: static position sense [1]. Static position and movement senses are both critical for accurate movement. For example, to correctly plan a reach, the brain needs an accurate estimate of the hand’s starting position. To monitor the movement in progress and make any needed corrections, the brain needs a good sense of movement. These sub-modalities share a common neural apparatus, including primarily spindles at non-extreme joint angles [1, 24], spinal pathway, and processing in the somatosensory cortices and cerebellum. Static position sense may arise from summation of background activity in spindles, while movement sense may arise from changes in spindle activity that occur in proportion to changes in muscle length [1]. Research in cats and monkeys suggests that position and movement information are simultaneously processed in the same neural networks [25, 26]. Thus, a disease or injury that impairs one sense is likely to impair the other. From a clinical standpoint, because of the limitations of tests involving movement, a movement-free clinical test of static position sense would have substantial advantages. Such a test would need to be standardized and objective, reliable, simple to apply, portable, inexpensive, and easy for patients to complete [27, 28].

Here we propose a novel proprioception measurement technique for the hand and fingers. The adaptive staircase method is an application of psychometric techniques to the measurement of static position sense, which allows us to estimate both proprioceptive sensitivity and bias [29]. Specifically, we place a tablet-style computer screen over the pronated hand with a white line presented at varying angular increments from the joint being tested. We measure proprioception in the plane of abduction/abduction at the metacarpophalangeal (MP) joint in 2^nd^ (index) finger of the right hand, chosen because of its importance for fine motor skill such as pinching small objects between the thumb and index finger. With each stimulus presentation, the subject reports whether he feels that the white line is left or right of his index fingertip. The procedure is related to the Parameter Estimation by Sequential Testing method developed by Taylor & Creelman [30] and recently applied in robot-assisted measurement of proprioception for research applications [31]. We compare the adaptive staircase method to carefully controlled versions of two common clinical tests, matching and PMDD, addressing three questions: (1) Which method has the best test-retest reliability? (2) Which method has the best inter-rater reliability? (3) Which method best reflects known proprioceptive changes associated with aging [10] and skilled movement [32, 33], i.e. construct validity?

## Methods

### Subjects

48 healthy right-handed adults (aged 18–82, 16 male) participated in the study. All three tests were performed in each session, with the order randomized. Study procedures were approved by the Indiana University Institutional Review Board. Subjects gave written informed consent and completed questionnaires about general health and activities: years playing a sport or musical instrument and average hours played per week. For all three techniques, subjects were seated in front of a table, with the apparatus on the table positioned centrally. The elbow was bent about 90° and slightly in front of the body, with the forearm resting on the table. The hand was pronated on the 25° apparatus, about 20cm in front of the body and centered with the trunk midline. The index finger was positioned at 55° to the subject’s trunk and the other fingers were slightly spread.

### Measurement procedures

#### Adaptive staircase technique

**Fig 1:**
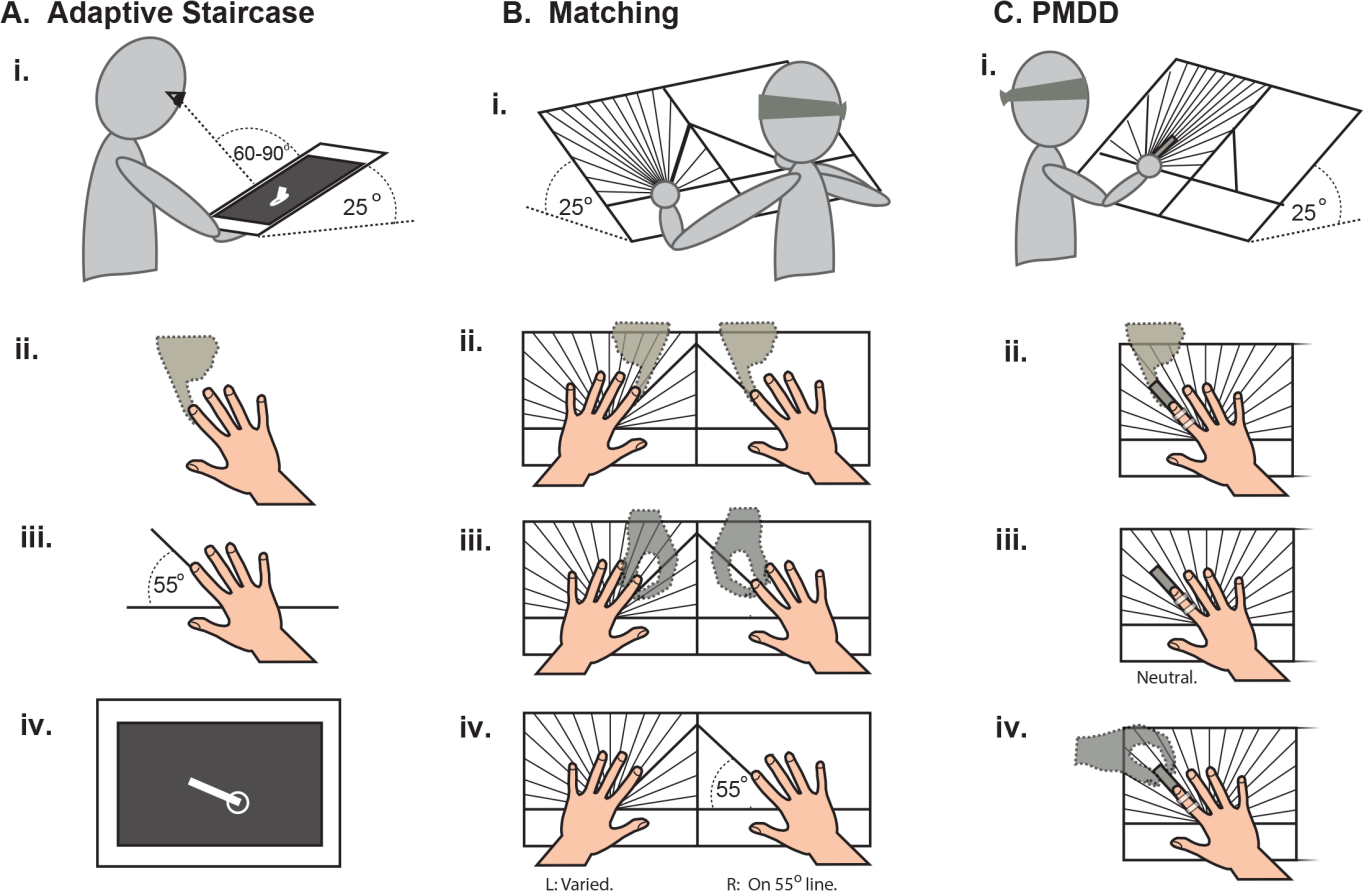
Setup procedures for each method. **A. i**. Subject positioning for the adaptive staircase method. Right hand placed on a 25° stand beneath the display screen. **ii.** The blindfolded subject was instructed to press firmly against the experimenter’s finger (dashed outline) and then relax to control for muscle thixotropy. **iii.** The hand was positioned in an outline drawn on the angled stand. The MP joint of the 2^nd^ finger was on a tactile marker with the index finger pointing along a 55° line and the other fingers slightly spread. **iv.** The display screen was placed over the subject’s hand and the blindfold removed. Subject was asked, “Do you feel like the end of the white line is to the left or to the right of your index fingertip?” **B. i.** Subject positioning for the matching method. Both hands placed in outlines on the angle board, inclined at 25°. Subject blindfolded throughout. **ii.** Controlling for muscle thixotropy in both index fingers. **iii.** The experimenter (dashed outlines) moved each index finger in turn with a variety of motions. **iv.** The experimenter placed the right index finger along the 55° line and the left in a variety of positions about the mirrored position. Subjects were instructed to “Move your left index finger so it mirrors your right index finger.” **C. i.** Subject positioning for the PMDD method. Right hand on the left half of the angle board, inclined at 25°. Index finger taped to a smooth stick. Subjects blindfolded throughout. **ii.** Controlling for muscle thixotropy. **iii.** Experimenter placed hand with fingers slightly spread and index finger in a neutral position. **iv.** Touching only the stick, experimenter moved subject’s index finger right or left (~0.062°/s) to a standard angular magnitude. Subject was asked, “Did I move your finger to the left or to the right?”

The blindfolded subject’s right hand was positioned on an angled stand (Fig 1Ai), with the MP joint of the 2^nd^ finger on a tactile marker and the index finger pointing along a 55° line (Fig 1Aiii). The experimenter was aided by the outline of a hand drawn on the stand. The subject was instructed to press firmly against the experimenter’s finger and then relax to ensure a consistent history of muscle contraction across subjects (a control for muscle thixotropy; Fig 1Aii). Finally, a tablet-style computer screen (13 x 9 inches, 8mm thick) was placed over the subject’s hand. The blindfold was removed and the subject instructed not to move the right hand further.

Subjects viewed the display of a custom MATLAB (R2013a MathWorks) program, a white line on a dark background (Fig 1Aiv). The line began directly over the tested joint (represented by a circle) and extended the length of the finger. Subjects were asked to report whether the end of the line was located to the right or left of their index fingertip. 6 staircases were completed for each application of this method. The first two staircases began 30° to the left and right of the true finger position (55°). If the subject’s response was correct (e.g. “left” when the white line was 30° to the left of the finger), the line moved 10° towards true finger position and the subject was again asked to report their perception. As the line approached true finger position, the choice of right or left became less obvious; eventually the subject felt the white line had overshot their finger and changed their reported direction. Whenever reported direction changed, the line reversed direction and step size decreased by half to yield more measurements near the subject’s perceptual boundary (the angle at which the subject is equally likely to report “left” or “right”). The first two staircases terminated when the subject had reversed direction 4 times. To increase the number of angles sampled near the subject’s actual perceptual boundary, a rough estimate of the boundary (mean of the last 4 angles tested in each staircase) and sensitivity (0.75 of the range of these 8 angles) in the subject’s perception was then calculated online. The subsequent 4 staircases were centered on the subject’s rough perceptual boundary, rather than true finger position, and began to the left or right (2 each) at an angular distance equal to the subject’s rough sensitivity. Initial step size for these staircases was 5°, and each staircase terminated when the subject had reversed direction 4 times. The mean number of proprioceptive estimates subjects made in the 6 staircases was 58 ±12. Subjects were given no feedback on their performance.

For each angle tested during the 6 staircases, the program calculated the number of responses obtained and the proportion that were “right” rather than “left.” This individual dataset was fit with a logistic regression model, chosen both because the data come from a binomial distribution and because this limits the predicted proportions to [0 1]. The 50% point of the fitted function was interpreted as the subject’s perceptual boundary, equivalent to the bias in the proprioceptive estimate of that joint’s position. The angular difference between the 25% and 75% points of the function was interpreted as a representation of the sensitivity in the subject’s proprioceptive estimate, with a smaller value reflecting greater sensitivity. Across individuals, the boundary and sensitivity obtained from the fitted function were nearly identical to those obtained from the mean and standard deviation of the angles at which the subject reversed direction (correlation r > 0.98, p < 10^−30^ for boundary and r > 0.92, p < 10^−20^ for sensitivity), suggesting the function fit was good. To assess whether a shortened version of this test would compare favorably with the other methods, we also re-analyzed this data using only the first 2 staircases.

#### Matching

To maximize consistency across subjects and experimenters, we devised a matching task that is less subjective than what might be done in a clinical setting, but still simple enough to be used clinically. Blindfolded subjects placed both hands on an inclined custom angle board (Fig 1Bi.): the right hand (reference hand) on the right panel and the left (indicator hand) on the left panel. The experimenter placed the hands such that the MP joint of each index finger was on a tactile marker where the lines intersect. Both panels had the outline of a hand drawn to aid the experimenter with positioning, plus a thick line at ±55° to indicate mirrored positions. Lines of different colors were drawn on the left panel in steps of 10, 5, 2.5, and 1.25°. After controlling for muscle thixotropy (Fig 1Bii), the experimenter moved each index finger in turn (Fig 1Biii) and then placed the right index finger along the 55° line (Fig 1Biv). Participants were then instructed to move the left index finger to mirror the right. The left finger’s final position was recorded in terms of angular deviation from a perfectly mirrored position (0°). This trial was repeated 10 times, with the experimenter moving and placing both index fingers before each trial to discourage subjects from using remembered positions. The subject’s proprioceptive bias and sensitivity were calculated as the mean and SD of the 10 errors.

#### Passive movement direction discrimination threshold (PMDD)

Rather than test the clinically-used PMDD, we devised a less-subjective version that could still be used clinically. PMDD was tested on the left panel of the inclined angle board (Fig 1Ci). Experimenters placed the blindfolded subject’s right hand so that the MP joint of the index finger was on the tactile marker where the lines intersect. To reduce extraneous sensations during passive movement, the index finger was taped to a smooth stick 1.5 x 0.75 x 10cm (Fig 1Cii). After controlling for muscle thixotropy (Fig 1Cii), the experimenter moved the finger 5° left or right. Subjects were asked to report perceived movement direction, then the finger was moved back to neutral. Experimenters touched only the stick during movements, to avoid giving the subject pressure cues. To control for movement speed, experimenters counted 2s between each 1.25° line on the board, equivalent to 0.625°/s [34]. For each tested angular magnitude, the subject experienced 6 movements: 3 to the left and 3 to the right, in randomized order. If the subject made a mistake at 5 degrees, angular magnitude was increased to 10°, and subsequently to 15° if mistakes were made at 10°. If the subject did not make mistakes at 5 degrees, angular magnitude was decreased to 2.5°, and then to 1.25° if no mistakes were made at 2.5°. The PMDD threshold was recorded as the smallest angle at which the subject did not make mistakes. The largest angular magnitude tested, due to biomechanical constraints, was 15°. The smallest angle tested, due to constraints of the angle board, was 1.25°.

### Statistical analysis

Test-retest reliability: we calculated intraclass correlation coefficients for each of 5 dependent variables (adaptive staircase boundary and sensitivity; matching mean and SD; PMDD threshold), comparing Session 1 to Session 2 [35]. Inter-rater reliability: we calculated intraclass correlation coefficients for each of the 5 dependent variables, comparing Rater 1 (YL) to Rater 2 (BS). Construct validity: we used a stepwise method to perform a multiple regression of each of the 5 dependent variables on 3 predictive terms: age, sport years x hours per week, music years x hours per week. We also computed correlations between each pair of tests: adaptive staircase boundary vs. PMDD threshold, adaptive staircase boundary vs. matching mean, matching mean vs. PMDD threshold, as well as equivalent pairs involving measures of sensitivity.

## Results

20 of the 48 subjects (age 18–73, 5 male) completed 2 experimental sessions each and were included in the test-retest analysis. The sessions were on average 14.9 ±10.7 days apart. The remaining 28 subjects completed one session each. For the 11 subjects (age 20–73, 3 male) included in the inter-rater analysis, measurements were performed twice in a single session by two different experimenters. At the end of each session, subjects were asked to rate their quality of sleep, fatigue from the session, and attention during the session on a scale from one to 10, with 10 being the most. Subjects reported an average (±SD) of 7.5±1.3, 3.3±2.2, and 8.2±1.1, respectively. All individual data is available in S1–S6 Files.

### Test-retest reliability

**Fig 2:**
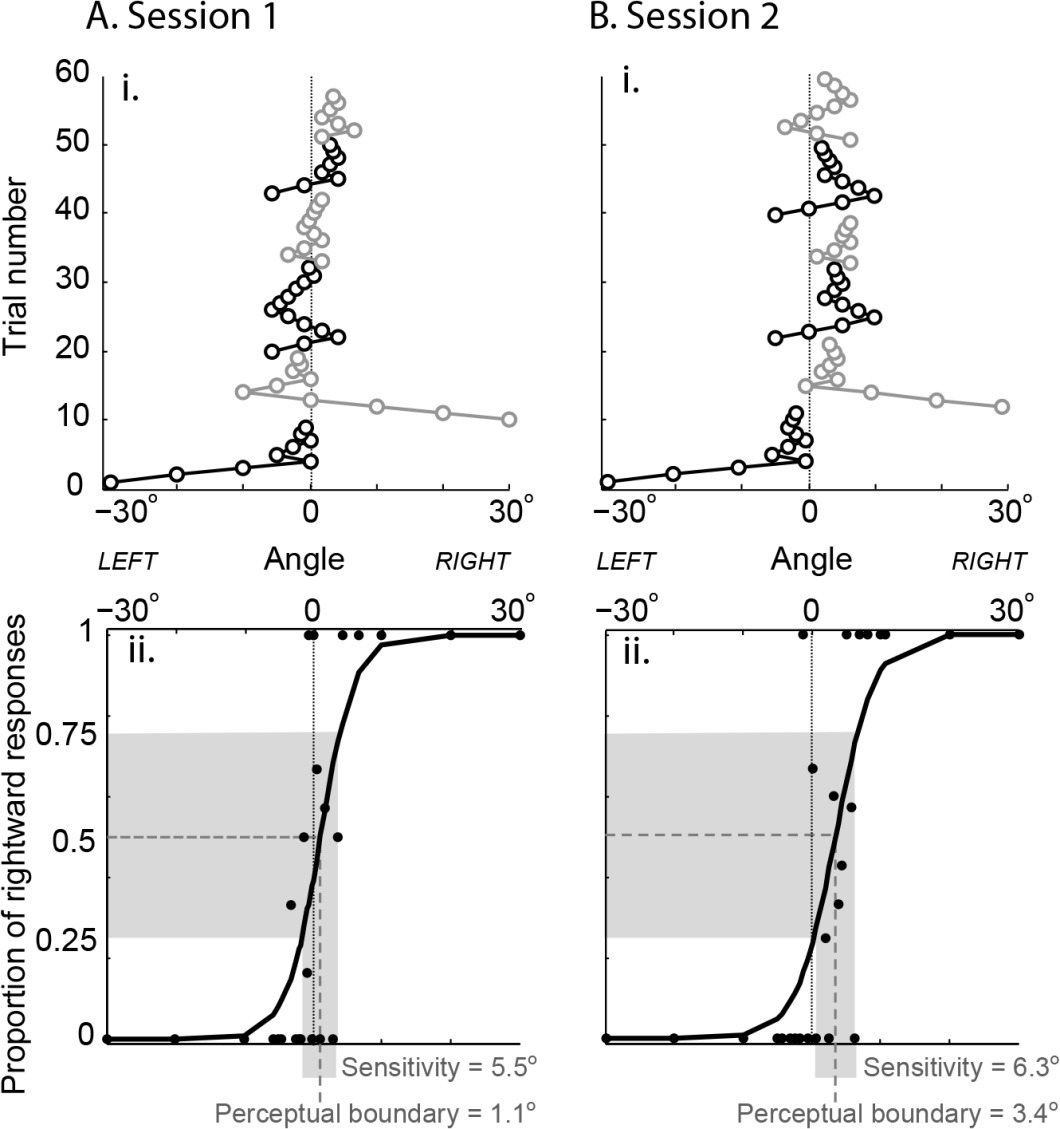
Adaptive staircase performance of a young subject (age 21) in two sessions conducted 10 days apart. **A. i.** Subject performance on 6 staircases in session 1: trial number vs. angle of the white line relative to true index finger angle (0°). Black: staircases that began with a leftward trial. Grey: staircases that began with a rightward trial. **ii.** Proportion of rightward responses at each tested angle (black circles) with fitted function. Perceptual boundary (proprioceptive bias) was 1.1°, proprioceptive sensitivity (width of grey shaded area on x-axis) was 5.5°. **B. i.** Subject performance 10 days later on 6 staircases. **ii.** Both perceptual boundary and sensitivity (3.4° and 6.3°, respectively) appear similar to the first session.

**Fig 3:**
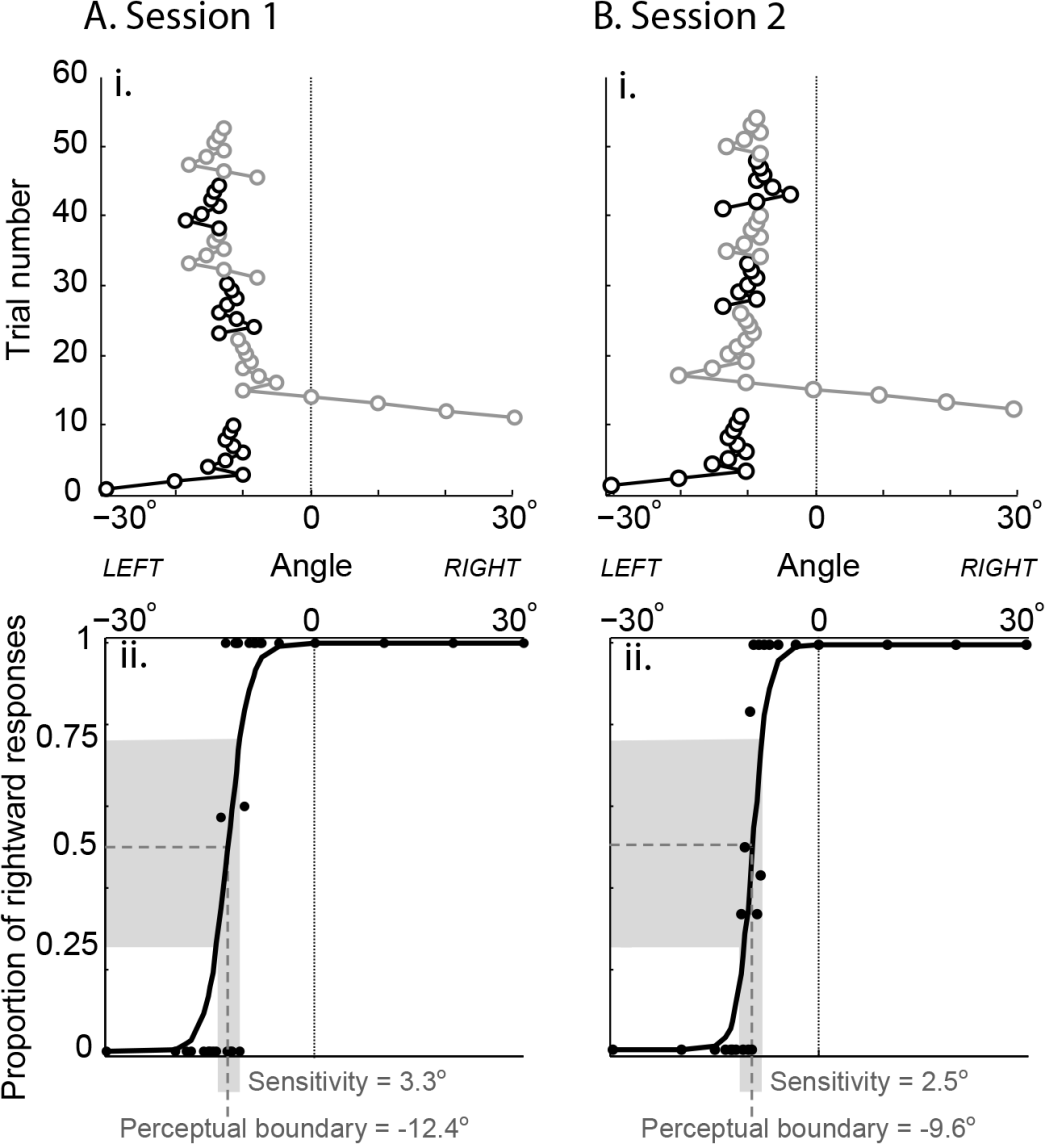
Adaptive staircase performance of an older subject (age 56) in two sessions conducted 7 days apart. **A. i.** Subject performance on 6 staircases in session 1: trial number vs. angle of the white line relative to true index finger angle (0°). Black: staircases that began with a leftward trial. Grey: staircases that began with a rightward trial. **ii.** Proportion of rightward responses at each tested angle (black circles) with fitted function. Perceptual boundary (proprioceptive bias) was -12.4°, proprioceptive sensitivity (width of grey shaded area on x-axis) was 3.3°. **B. i.** Subject performance 7 days later on 6 staircases. **ii.** Both perceptual boundary and sensitivity (-9.6° and 2.5°, respectively) appear similar to the first session.

Perceptual boundary (proprioceptive bias) as measured by the adaptive staircase technique showed the most stability. This was evident across the age range of subjects. Young subjects typically showed very small proprioceptive biases (e.g., Fig 2), while older subjects often showed larger proprioceptive biases (e.g., Fig 3). The spread of proprioceptive estimates (sensitivity) with the adaptive staircase method also appeared consistent across sessions, but no difference between older and younger adults was evident.

**Fig 4:**
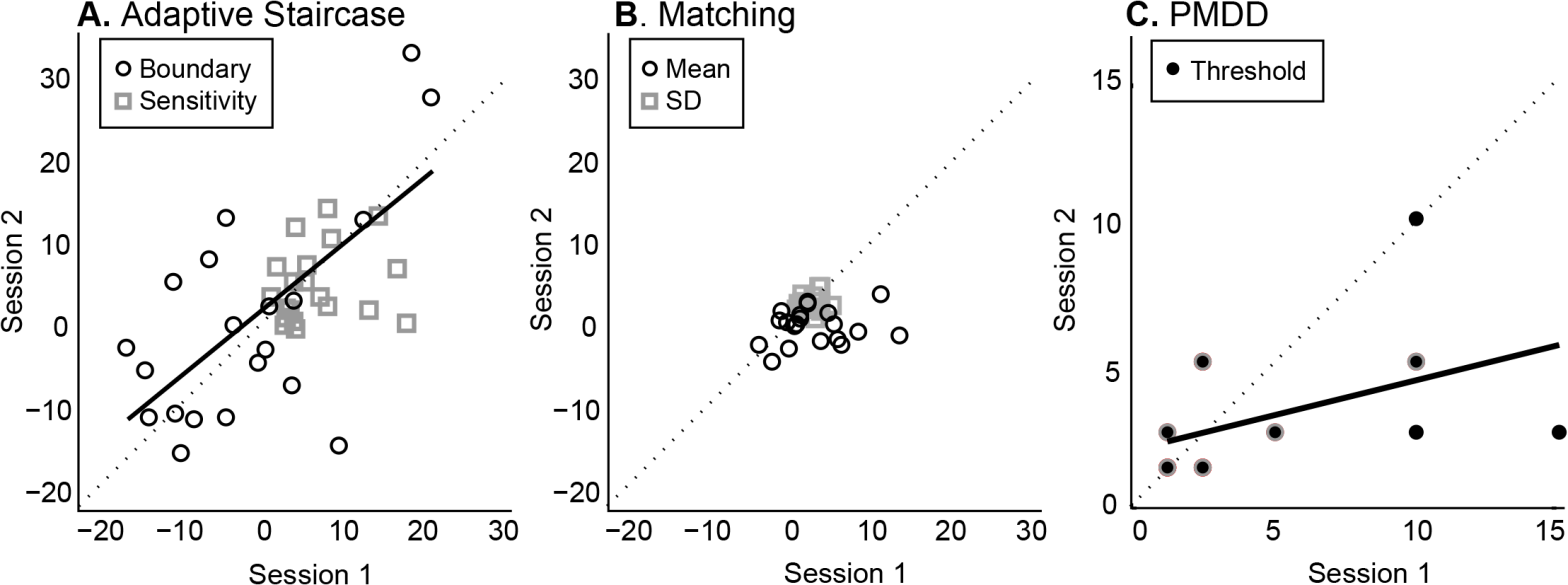
Group data on test-retest reliability (N = 20). **A.** Adaptive staircase measurement yielded a strong positive correlation between subject performance in Session 1 and 2 for perceptual boundary (proprioceptive bias, black circles). Best fit line (solid) is close to the 1:1 line (dashed), suggesting no overall difference between the two sessions. Proprioceptive sensitivity (grey squares) was not significantly correlated across sessions. **B.** Matching did not produce a significant correlation across sessions for either mean (reflects proprioceptive bias, black circles) or SD (reflects proprioceptive sensitivity, grey squares). **C.** PMDD showed a positive correlation across sessions for threshold (black filled circles with grey outlines reflect more than one subject), but the shallow slope indicates that thresholds were smaller overall in session 2 compared to session 1.

This pattern was observed in the group data as well. Proprioceptive bias as assessed with the adaptive staircase technique yielded the strongest test-retest reliability, whether the full 6 staircases were analyzed (ICC R = 0.62, p = 0.001; Fig 4A) or a short version including only the first two staircases (ICC R = 0.59, p = 0.002). One subject is an outlier according to quartile analysis, but as ICC is still significant after exclusion (ICC R = 0.52, p = 0.008), this subject does not appear to drive the relationship. Proprioceptive sensitivity, however, was not as stable (ICC R < 0.22, p > 0.17), which may be accounted for by the lack of spread in this population. The matching method appears the least stable (bias ICC R = -0.07, p > 0.6; sensitivity ICC R = 0.18, p > 0.22; Fig 4B), with the PMDD method in between (ICC R = 0.34, p = 0.007; Fig 4C). The PMDD data also suggests a learning effect, with thresholds decreasing for nearly all subjects in their second session (Fig 4C).

### Inter-rater reliability

**Fig 5:**
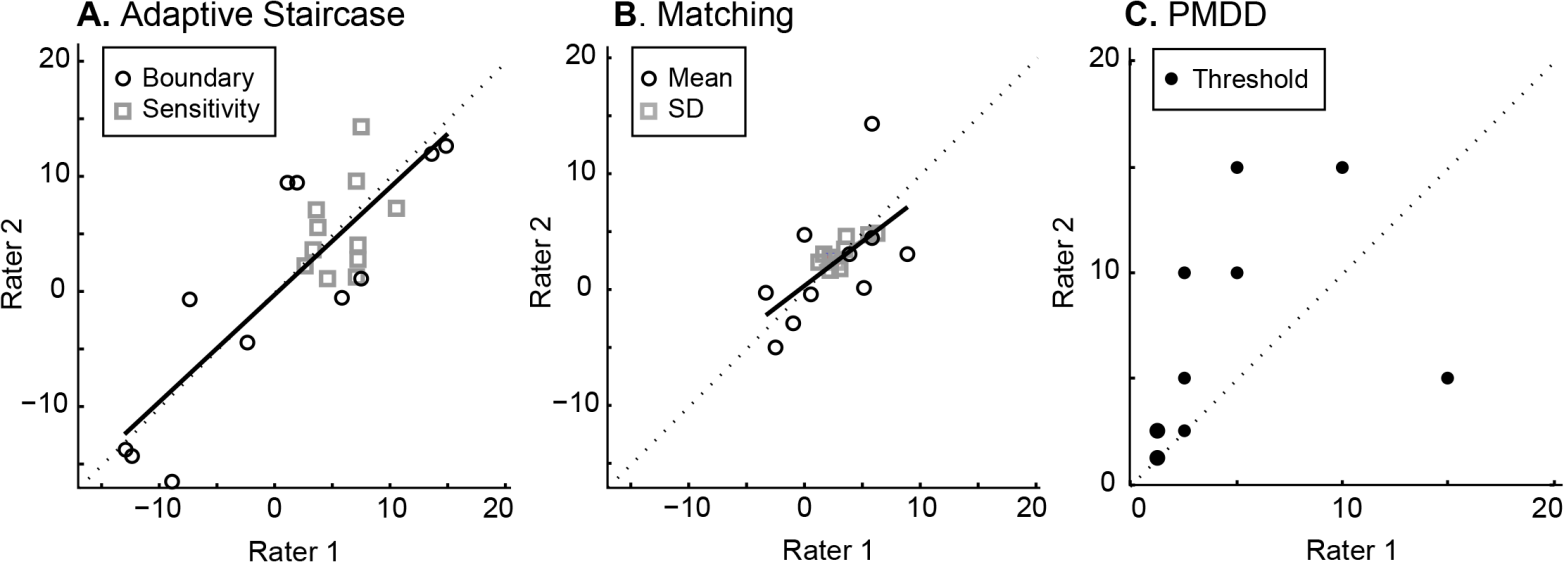
Group data on inter-rater reliability (N = 11). **A.** There was strong agreement across raters for perceptual boundary (proprioceptive bias, black circles) measured by the adaptive staircase method. Best fit line (solid) is close to the 1:1 line (dashed), suggesting no overall difference between the two raters. Proprioceptive sensitivity (grey squares) was not significantly correlated across raters. **B.** Both mean and SD of matching estimates were correlated across raters. **C.** Inter-rater reliability for PMDD was poor, with Rater 2 determining larger thresholds than Rater 1. Black filled circles with grey outlines reflect more than one subject.

Adaptive staircase measurement of perceptual boundary also yielded the greatest inter-rater reliability (Fig 5A). This was the case for both the long (ICC R = 0.86, p<0.001) and short (ICC R = 0.76, *p* = 0.0015) versions of the test. However, inter-rater reliability was poor for proprioceptive sensitivity with both versions (ICC R<0.20, p>0.13), again perhaps due to the small range of sensitivity in this healthy population. Matching showed the next highest inter-rater reliability for proprioceptive bias as assessed by the mean of 10 trials (ICC R = 0.60, *p* = 0.016), and the highest for proprioceptive sensitivity as assessed by the SD of 10 trials (ICC R = 0.77, p = 0.0012; Fig 5B). In contrast the PMDD measurement yielded weak inter-rater reliability (ICC R = 0.40, p = 0.089; Fig 5C). Clear differences between the raters are evident, with Rater 2 finding higher thresholds than Rater 1 (Fig 5C).

### Construct validity

**Fig 6:**
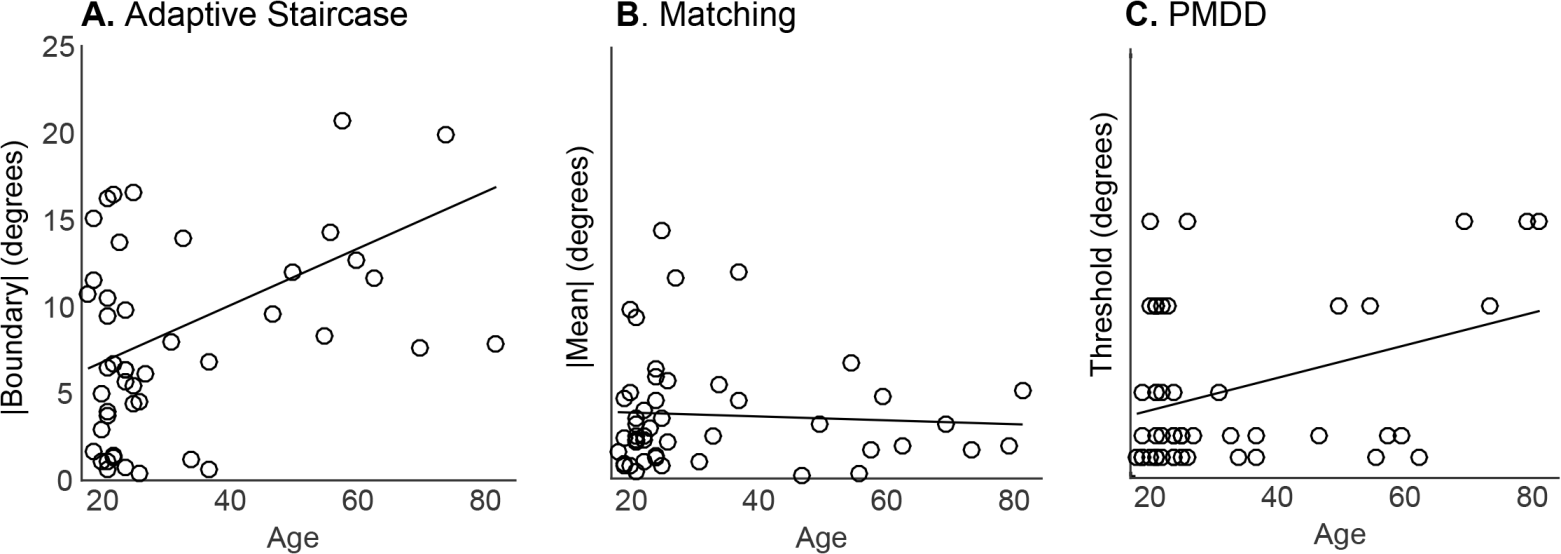
Group data on construct validity, with regression lines (N = 48). **A.** Perceptual boundary (absolute value) as measured with the adaptive staircase method showed a positive relationship with subject age, with older subjects having larger proprioceptive biases. **B.** Mean matching estimate (absolute value) is not predicted by subject age. **C.** PMDD threshold was also positively predicted by subject age, with older subjects tending to have higher thresholds.

Stepwise regression on age, sport years x hours per week, and music years x hours per week yielded only age as a significant predictive term for any of the three techniques. Age most strongly predicted proprioceptive bias as assessed by the adaptive staircase perceptual boundary (long method R^2^ = 0.17, p = 0.003; short method R^2^ = 0.13, p = 0.012). This is illustrated at the individual level by Figs 2 and 3; older subjects tended to have larger proprioceptive biases than younger subjects (Fig 6A), a difference that was maintained across multiple sessions. No significant predictors were found for proprioceptive sensitivity with the adaptive staircase method. PMDD threshold was also predicted by age (R^2^ = 0.13, p = 0.011, Fig 6C). However, no significant predictors were found for either mean or SD of the 10 estimates obtained in the matching method (Fig 6B).

There was a tendency for adaptive staircase boundary to be correlated with PMDD threshold (R = 0.21, p = 0.081) and for adaptive staircase sensitivity to be correlated with matching SD (R = 0.21, p = 0.079), which may support the construct validity of the adaptive staircase method. There were no other significant or trend correlations across methods, using either proprioceptive bias or sensitivity measures (p’s > 0.1).

## Discussion

**Fig 7:**
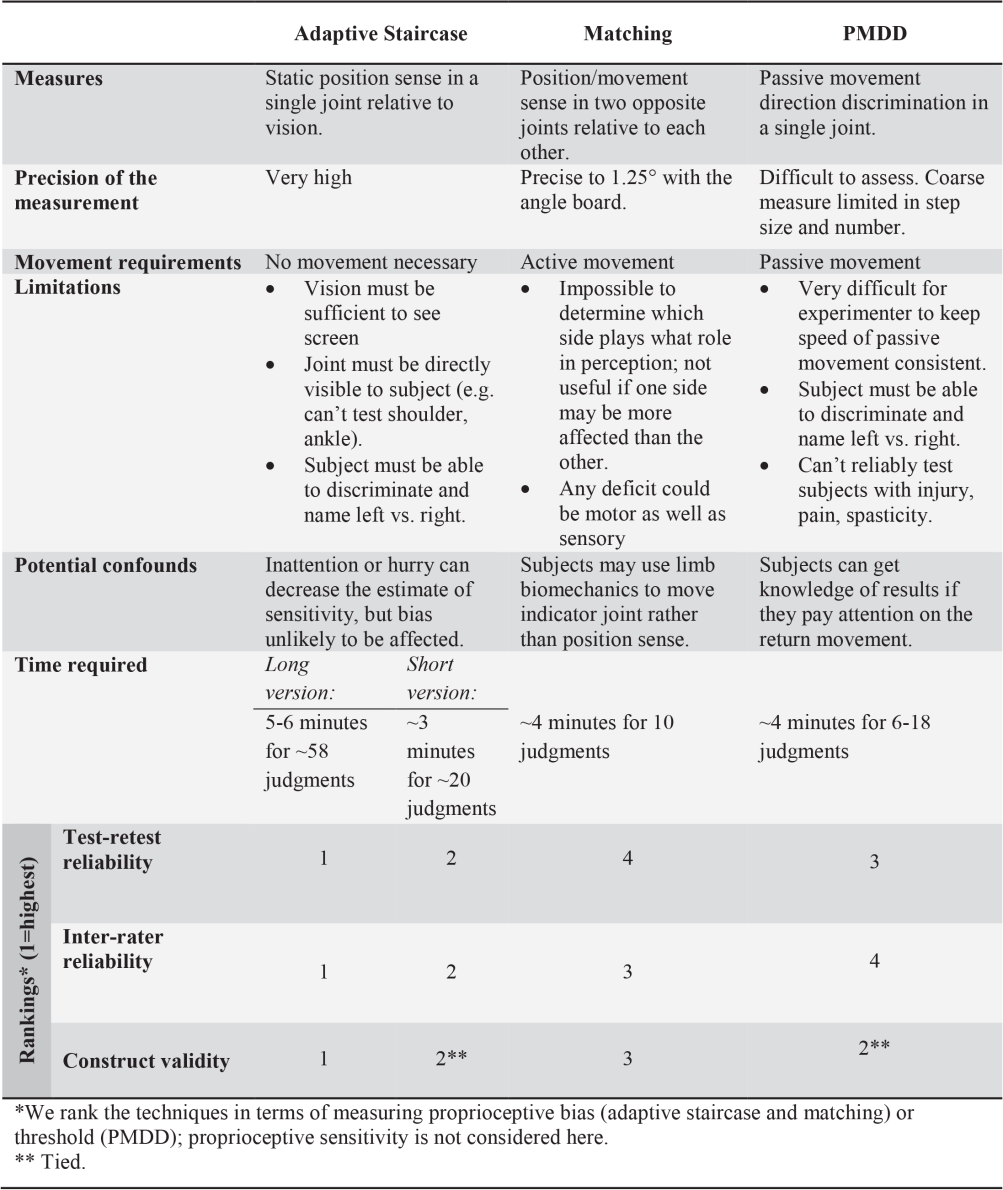
Comparison of the three measurement techniques.

We compared a novel technique for measuring proprioception (adaptive staircase method) with modified versions of two tests already used clinically (matching and PMDD). We found substantial differences in test-retest reliability, inter-rater reliability, and construct validity (Fig 7). Proprioceptive bias assessed by adaptive staircase method was strongest in all three analyses. A shortened version (~20 trials instead of ~60) performed nearly as well in all areas, and better than matching or PMDD. The matching method may be the weakest of those tested, having statistically significant inter-rater reliability only. The PMDD trended toward test-retest and inter-rater reliability, although with noticeable biases between days and raters, and was significantly predicted by age, indicating good construct validity.

The adaptive staircase method is an application of psychophysical techniques to proprioception. As a scientific discipline, psychophysics investigates the connection between physical stimuli and subjective responses via the psychometric function [29]. Responses are often obtained using a staircase procedure: the stimuli are presented in ascending or descending order of strength, with the subject choosing one of two options for each stimulus (two alternative forced choice), e.g. left or right. A series of staircases can then be used to create the psychometric function. This technique has historically been used to test visual perception, e.g. to assess the perception of color, brightness, and other properties. In recent years, this method has been applied to proprioception by researchers of motor control. E.g., with the subject unable to see his hand, a robot moves the hand in an adaptive staircase algorithm, with subjects judging final hand position relative to a visual marker or to proprioceptive straight-ahead [36, 37], or judging which direction the robot moved them [31, 38]. While these techniques are powerful, they require costly equipment.

### Strengths and limitations of the techniques

Assessing proprioception is a complex endeavor no matter what method is chosen. It cannot be measured directly, but must be based on the subject’s responses. In addition, proprioception is not one homogenous sensory modality. Rather, it consists of sub-modalities, e.g. static position sense, passive motion sense, sense of effort [1]. The adaptive staircase assesses static position sense with the hand unmoving, while matching assesses static position sense after movement. The PMDD method, in contrast, assesses passive motion sense. All three measures were applied with the finger at 55° to the subject’s trunk, about 20cm in front of and centered with the trunk. The hand was pronated, fingers slightly spread, forearm resting on the table, and elbow bent about 90° and slightly in front of the body. We did not test generalizability to other postures or finger angles, seeking instead to find a single posture and angle that could be tested easily and accurately. Proprioceptive bias and sensitivity as measured in these conditions may not generalize to other arm postures, as finger perception can be affected by forearm muscles [1], or to extreme finger angles, as joint receptors can bias perception in these circumstances [24]. It should also be noted that we asked subjects to make judgments about their fingertip position, not their joint angle. The brain is thought to be better at perceiving the endpoint effector than individual joints [24], so proprioceptive estimates generated this way will not necessarily agree with measures asking subjects to estimate joint angles explicitly.

#### Adaptive staircase method

Strongest in terms of test-retest reliability, inter-rater reliability, and construct validity, this method assesses static position sense in a single unmoving finger. Once the subject’s hand is positioned, the test is automated and not dependent on subjective judgments by the experimenter, which is advantageous in terms of training and flexibility. This method requires sustained attention from subjects, which was a challenge for the 5–6 minute long version with 6 staircases. However, analysis indicates that shortening the test to 2 staircases (2–3 minutes including setup) would yield results very close to the long version, and would still be stronger than matching or PMDD. The shortened version may thus be preferable in clinical settings.

An added strength of the adaptive staircase method is the independent estimation of proprioceptive bias and sensitivity. In terms of functional dexterity, bias measured during the adaptive staircase test is expected to correspond to bias errors in the subject’s movements: e.g., when relying on proprioception to pick up a small object like a pen with the index finger and thumb, a subject with high sensitivity but a large leftward bias might direct his index finger too far to the right of the pen. If he attempted this task many times, because of his high sensitivity he would be too far to the right of the pen very consistently. In contrast, a subject with low bias and low sensitivity would simply be more variable at the task: sometimes his index finger would end up exactly on the pen, but other times he would be to the left or to the right. Bias and sensitivity deficits could also be combined in any proportion. Both would impair functional dexterity and potentially be detrimental to the subject’s quality of life; the primary reason for measuring both of these parameters is that some clinical populations may be impaired on only one, others on both, etc., so a test that only measures sensitivity could miss the proprioceptive deficits in a population that has impairment only in bias. These predictions for functional dexterity need to be tested in clinical populations, which are likely to have a wider spread of bias (and potentially sensitivity) than the healthy subjects in the present study.

#### Matching

Assesses static position sense after movement. Although more sophisticated versions of this test have been used effectively in proprioception research [17, 39], it does not translate well to the clinic, as researchers often use their own custom apparatus to increase accuracy [17, 21]. Despite the popularity of this method in clinical settings, its reliability and validity have rarely been investigated [40]. The technique did poorly in our tests of test-retest reliability and construct validity, but did show evidence of inter-rater reliability, perhaps because we used the angle board to reduce subjectivity. There does appear to be a learning effect, however, with subjects’ means ranging from -5 to 12° in session 1 but clumped closely around 0° in session 2. We also found a potential confound related to body biomechanics; because the right index finger’s position was close to neutral, relaxing the left hand could place the left index finger in an approximately mirrored position, a strategy that several subjects reported using. A major limitation of the matching method is that it is impossible to determine which side plays what role in perception; i.e., it cannot be determined whether one side is more affected than the other [36], and any deficit could be motor as well as sensory. There may also be interactions between the brain’s proprioceptive estimates of each index finger; the noticeably smaller range in error for matching compared to the other methods suggests that involving both hands may yield a perceptual advantage, potentially an added complication for a clinical test. An additional limitation is the requirement for active movement. Any patient with movement difficulties, pain, impaired manual dexterity, etc, cannot be reliably tested this way.

#### PMDD

Reflects a subject’s sense of passive movement [41] and is a widely used clinical method. However, the technique is highly subjective as applied in the clinic. Movement magnitude and speed are poorly standardized, yet both parameters influence movement sense [42]. Correlation between PMDD and clinical outcome is only low to moderate [42]. In our tests, PMDD had good construct validity (strong relationship with subject age), but test-retest and inter-rater reliability did not reach significance. Although we used the angle board to reduce subjectivity and standardized movement extent and speed, the trained experimenters still found it difficult to keep movement speed constant. The method usually applied clinically, without any of these controls, is likely even less reliable. Despite our care, several subjects spontaneously reported that they gained knowledge of results by paying attention on the return movements. The required passive movement may also be problematic with some populations: it can’t reliably be used in patients with injury, pain, or spasticity. It should be noted that adaptive staircase procedures can be applied to movement sense, as has been done in research applications [31, 38]. However, performing the large number of trials needed to build a psychometric function, at the slow speed required, would be prohibitively time consuming for a clinical test.

### Other considerations

#### Bias vs. sensitivity in static position sense measurement

For both the adaptive staircase and matching methods, only proprioceptive bias yielded significant results. With one exception (inter-rater reliability for matching), no relationships involving the spread of proprioceptive estimates (analogous to sensitivity) were found. While it is possible that proprioceptive sensitivity is not as useful a measure as bias, this cannot be concluded definitively from our data. Sensitivity was tightly grouped across subjects in our data; there was not enough spread to determine a correlation in most cases. This could be due to our focus on healthy subjects; it is possible that a patient group with impaired proprioception would have a larger spread in sensitivity. Well-controlled studies in patient populations are needed to determine whether measurements of proprioceptive sensitivity have reliability and validity in these groups.

#### Skilled movement and comparison across methods

Stepwise regression did not identify music or sport involvement as a predictor for proprioception with any of the techniques. This does not necessarily mean these factors do not affect proprioception; our subject pool could again be responsible. Although we were able to recruit healthy subjects with a wide range of age, only a handful had substantial music or sport involvement. The non-uniform distribution in these variables could account for the lack of relationship with proprioception.

Correlations calculated between the three methods identified only two trends: adaptive staircase boundary correlated with PMDD threshold, and adaptive staircase sensitivity correlated with matching SD, supporting the construct validity of the adaptive staircase method. Interestingly, matching and PMDD did not approach a significant correlation with each other. This is perhaps surprising considering that these methods are commonly used in the clinical setting, but considering the limited evaluation of the clinimetric properties of these tests [21, 22], and their poor test-retest reliability, inter-rater reliability, and construct validity in the healthy population tested here, there may be reason to question the usefulness of these tests in clinical settings.

### Conclusions

We have introduced an adaptive staircase technique in healthy adults to measure proprioception in the hand and fingers. This method requires no movement but has strong test-retest and inter-rater reliability as well as construct validity. It is simple to use and portable, and could easily be translated into a clinical tool. Both proprioceptive bias and sensitivity are estimated, which could be useful in tailoring rehabilitation to specific proprioceptive deficits. Studies that include other finger joints and the wrist are needed to determine how generalizable the technique may be, and studies in patient populations are needed to examine the utility of this method when proprioception is impaired.

## Supporting Information

S1 File All session 1 data. Tab-delimited text file of de-identified individual data from 48 subjects used to evaluate construct validity. 48 columns, each representing a subject, by 27 rows, each representing a variable listed in Variables1.txt.
(TXT)

S2 File Test-retest session 1 data. Tab-delimited text file of de-identified individual data from the first session of 20 subjects used to evaluate test-retest reliability. 20 columns, each representing a subject, by 27 rows, each representing a variable listed in Variables1.txt.
(TXT)

S3 File Test-retest session 2 data. Tab-delimited text file of de-identified individual data from the second session of 20 subjects used to evaluate test-retest reliability. 20 columns, each representing a subject, by 27 rows, each representing a variable listed in Variables1.txt.
(TXT)

S4 File Inter-rater data. Tab-delimited text file of de-identified individual data from the 11 subjects used to evaluate inter-rater reliability. 11 columns, each representing a subject, by 18 rows, each representing a variable listed in Variables2.txt.
(TXT)

S5 File Variable names in S1–S3 Files. List of the variable names represented by rows in All_S1.dat, Test-retest_S1.dat, and Test-retest_S2.dat.
(TXT)

S6 File Variable names in S4 File. List of the variable names represented by rows in Inter-rater.dat.
(TXT)
